# Identification and validation of hub microRNAs dysregulated in esophageal squamous cell carcinoma

**DOI:** 10.18632/aging.103245

**Published:** 2020-05-15

**Authors:** Chen Sang, Ce Chao, Min Wang, Youpu Zhang, Guanghua Luo, Xiaoying Zhang

**Affiliations:** 1 Department of Cardiothoracic Surgery, The Third Affiliated Hospital of Soochow University, Changzhou 213003, China; 2 Comprehensive Laboratory, The Third Affiliated Hospital of Soochow University, Changzhou 213003, China

**Keywords:** esophageal squamous cell carcinoma, bioinformatics, hub miRNAs, tumorigenesis

## Abstract

Esophageal squamous cell carcinoma (ESCC) is one of the deadliest cancers worldwide, and its morbidity is exacerbated by the lack of early symptoms. Bioinformatics analyses enable discovery of differentially expressed genes and non-protein-coding RNAs of potential prognostic and/or therapeutic relevance in ESCC and other cancers. Using bioinformatics tools, we searched for dysregulated miRNAs in two ESCC microarray datasets from the Gene Expression Omnibus (GEO) database. After identification of three upregulated and five downregulated miRNAs shared between databases, protein-protein interaction (PPI) network analysis was used to identify the top 10 hub-gene targets. Thereafter, a miRNA-gene interaction network predicted that most hub genes are regulated by miR-196a-5p and miR-1-3p, which are respectively upregulated and downregulated in ESCC. Functional enrichment analyses in the GO and KEGG databases indicated the potential involvement of these miRNAs in tumorigenesis-related processes and pathways, while both differential expression and correlation with T stage were demonstrated for each miRNA in a cohort of ESCC patients. Overexpression showed that miR-196a-5p increased, whereas miR-1-3p attenuated, proliferation and invasion in human ESCC cell lines grown in vitro. These findings suggest miR-196a-5p and miR-1-3p jointly contribute to ESCC tumorigenesis and are potential targets for diagnosis and treatment.

## INTRODUCTION

Esophageal carcinoma ranked as the ninth most common cancer worldwide in 2018, with both new cases and deaths exceeding 500,000 and a 5-year survival of ~20% [1]. Esophageal squamous cell carcinoma (ESCC) is the predominant esophageal carcinoma histological subtype in China, and lack of early disease signs contributes to its high prevalence and morbidity [2]. Attending to the pressing need for more accurate biomarkers for early diagnosis and treatment of ESCC and other cancers, much attention is being devoted to the study of microRNAs (miRNAs), which have shown to influence tumor development by dynamic post-transcriptional regulation of gene expression [3, 4]. Research has shown that several miRNAs are differentially expressed in ESCC and may contribute to its development. For example, upregulated expression of miR-502 and miR-26b regulates ESCC cell proliferation and tumor progression by promoting the phosphorylation of AKT and controlling cell cycle transitions, respectively [5, 6]. In contrast, two miRNAs downregulated in ESCC function as tumor suppressors; miR-302b represses the expression of erb-b2 receptor tyrosine kinase 4 (ErbB4) whereas miR-134 downregulates PLXNA1 and blocks the mitogen-activated protein kinase (MAPK) signaling pathway [7, 8]. However, given the heterogeneous nature of ESCC, other relevant transcripts among the many differentially expressed miRNAs are likely to affect ESCC pathogenesis.

By allowing parallel processing of massive quantities of high-throughput sequencing data, bioinformatics plays now a very important role in the exploration of disease mechanisms and is providing valuable insights into the landscape of differentially expressed genes and key regulatory non-protein-coding RNAs (ncRNAs) involved in ESCC development and metastasis [9–11]. In this study we applied bioinformatics tools to interrogate two GEO datasets with the goal of identifying differentially expressed miRNAs (DEMs) in ESCC. Analysis of miRNA target genes is of primary importance. MiRTarBase contains the target genes of miRNA that have been verified by experiments, and we can further analyze their differential expression level in tumors through UALCAN. DAVID can help us carry out Gene Ontology (GO) and Kyoto Encyclopedia of Genes and Genomes (KEGG) pathway analyses to point out the signaling pathways that these target genes may be involved in. Cytoscape software can helps us visualize miRNA-target gene pairs. miRNACancerMAP can also analyze the signal pathways that miRNA may be involved in through published literature. Through the application of the above database tools, we unmasked two DEMs, each one potentially regulating several hub genes dysregulated in ESCC, and verified their expression and prognostic value on ESCC. After expression and clinicopathological correlation analyses on internal ESCC cases, we asserted through miRNA mimics transfection significant and opposing effects of the two DEMs on the proliferation and migratory ability of human ESCC cell lines cultured in vitro. Our findings may advance further research to define the prognostic value of these miRNAs and their therapeutic potential to block ESCC progression.

## RESULTS

### Screening and identification of differentially expressed miRNAs and target genes in ESCC

**Figure 1:**
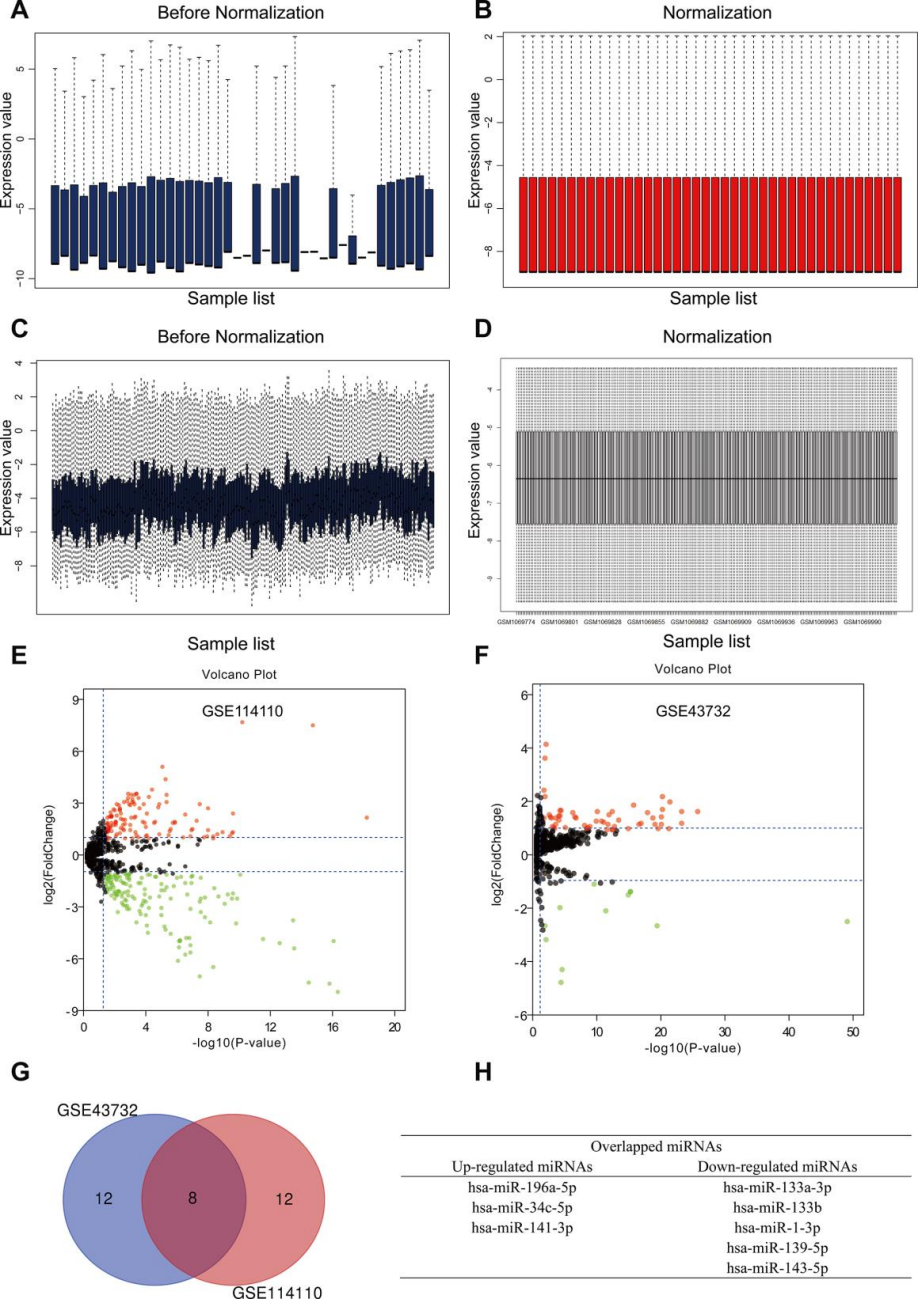
**Identification of differently expressed miRNAs (DEMs) in esophageal squamous cell carcinoma (ESCC).** (**A, B**) GSE114110 data before and after normalization. (**C, D**) GSE43732 data before and after normalization. (**E, F**) Volcano plots of DEMs in GSE114110 and GSE43732, respectively. Black dots represent genes equally represented between ESCC and normal samples. Red and green dots represent upregulated and downregulated miRNAs, respectively. Volcano plots showing all DEMs. |log2FC| ≥ 1 and *P* < 0.05 were set as cut-off criteria. (**G**) Venn diagram analysis showing the top 10 upregulated and downregulated miRNAs in the two GEO datasets. (**H**) Identification of three upregulated and five downregulated miRNAs overlapping between both GEO datasets.

To identify differentially expressed miRNAs (DEMs) in ESCC, data from two independent miRNA expression arrays (GSE114110 and GSE43732) [12, 13] were downloaded from the GEO database and normalized using the limma software package of R language (Figure 1A–1D). According to the thresholds set (*p* < 0.05 and log2FC ≥ 1), 277 and 68 DEMs were found in GSE114110 and GSE43732, respectively. Subsequently, we obtained the top 20 DEMs in each dataset (Tables 1 and 2 and Figure 1E and 1F), and identified through Venn diagram analysis three upregulated and five downregulated DEMs held in common between the two datasets (Figure 1G and 1H). Based on these eight DEMs, miRNA-target gene interactions were evaluated using the experimentally validated miRTarBase database [14]. A total of 468 and 753 possible target genes, respectively, were thus identified for the three upregulated and five downregulated hub miRNAs.

**Table 1:** Top twenty DEMs in ESCC tumor tissues (GSE114110).

**row.names(tT)**	**logFC**	**AveExpr**	**P.Value**	**adj.P.Val**
hsa-miR-196a-5p	7.79797876	-3.387916534	7.57248E-11	1.29902E-08
hsa-miR-196b-5p	7.575340044	-3.197327593	2.50005E-15	9.43519E-13
hsa-miR-34c-5p	5.20100599	-4.943344858	1.06583E-05	0.000209502
hsa-miR-431-3p	4.47689702	-5.878727839	6.31206E-06	0.000132343
hsa-miR-4697-5p	3.885631685	-3.253089306	5.24783E-05	0.000853678
hsa-miR-141-3p	3.657871024	3.344289289	0.00046881	0.005332609
hsa-miR-378b	3.64168965	-4.821279397	0.000532989	0.005951188
hsa-miR-3174	3.63373916	-6.004442677	0.001614964	0.015086324
hsa-miR-3934	3.596816037	-4.867145834	0.001003633	0.010062287
hsa-miR-31-5p	3.589730214	1.230595469	0.000715657	0.00776118
hsa-miR-133a	-7.835809282	-6.021408944	6.15255E-17	5.80493E-14
hsa-miR-4328	-7.334595008	-6.621866677	2.06269E-16	9.73075E-14
hsa-miR-4770	-7.286226861	-7.135112011	4.58049E-15	1.44056E-12
hsa-miR-133b	-6.919795614	-2.695254415	4.05741E-08	1.8674E-06
hsa-miR-1	-6.415395245	-0.758690446	6.30884E-09	4.1051E-07
hsa-miR-143-5p	-6.035861661	-4.104229206	1.04533E-06	2.85876E-05
hsa-miR-30a-3p	-5.6534869	-4.240228256	3.81629E-07	1.16151E-05
hsa-miR-139-5p	-5.549525869	-4.783094569	1.67798E-07	5.8636E-06
hsa-miR-136-3p	-5.307706051	-7.48286509	3.56046E-14	9.59797E-12
hsa-miR-381	-5.226057181	-5.550279909	9.24308E-07	2.56496E-05

DEMs (Differentially expressed miRNAs).

**Table 2:** Top twenty DEMs in ESCC tumor tissues (GSE43732).

**row.names(tT)**	**logFC**	**AveExpr**	**P.Value**	**adj.P.Val**
hsa-miR-4700-3p	4.207121008	-6.919626701	0.019593424	0.057045772
hsa-miR-7-2-3p	3.697998047	-7.681909385	0.029038083	0.078855711
hsa-miR-141-3p	2.592871786	-4.638740689	0.029009219	0.078855711
hsa-miR-369-3p	2.480429667	-6.264850093	0.036991106	0.096888029
hsa-miR-488-3p	2.268432275	-6.559615961	0.027596813	0.076105496
hsa-miR-196a-5p	2.26773491	-6.130951651	1.22E-20	1.36E-18
hsa-miR-450a-5p	2.050135385	-6.40331426	7.93E-22	1.41E-19
hsa-miR-34c-5p	1.957777329	-6.66978844	3.38E-16	1.67E-14
hsa-miR-944	1.777326902	-6.724237795	1.17E-20	1.36E-18
hsa-miR-301b	1.747287368	-7.403315465	0.0000872	0.000560884
hsa-miR-200b-3p	-4.693526632	-4.537349541	0.0000713	0.000469247
hsa-miR-200c-3p	-4.201274199	-4.380604976	0.0000636	0.000427741
hsa-miR-205-5p	-3.087222059	-4.524338153	0.014865414	0.045518923
hsa-miR-375	-2.562634964	-5.417712355	7.22E-20	5.35E-18
hsa-miR-139-5p	-2.424075348	-6.147630342	1.82E-49	1.62E-46
hsa-miR-133b	-2.012607522	-5.484360223	6.85E-12	1.79E-10
hsa-miR-1	-1.889046892	-5.399187166	0.00011331	0.000689172
hsa-miR-133a	-1.43052223	-5.940470029	2.98E-15	1.2E-13
hsa-miR-143-5p	-1.305017409	-5.457683389	1.14E-15	5.33E-14
hsa-miR-30a-3p	-1.282380928	-5.746203958	1.2E-15	5.33E-14

DEMs (Differentially expressed miRNAs).

### Gene Ontology (GO) and Kyoto Encyclopedia of Genes and Genomes (KEGG) pathway analyses of hub miRNAs’ target genes

**Figure 2:**
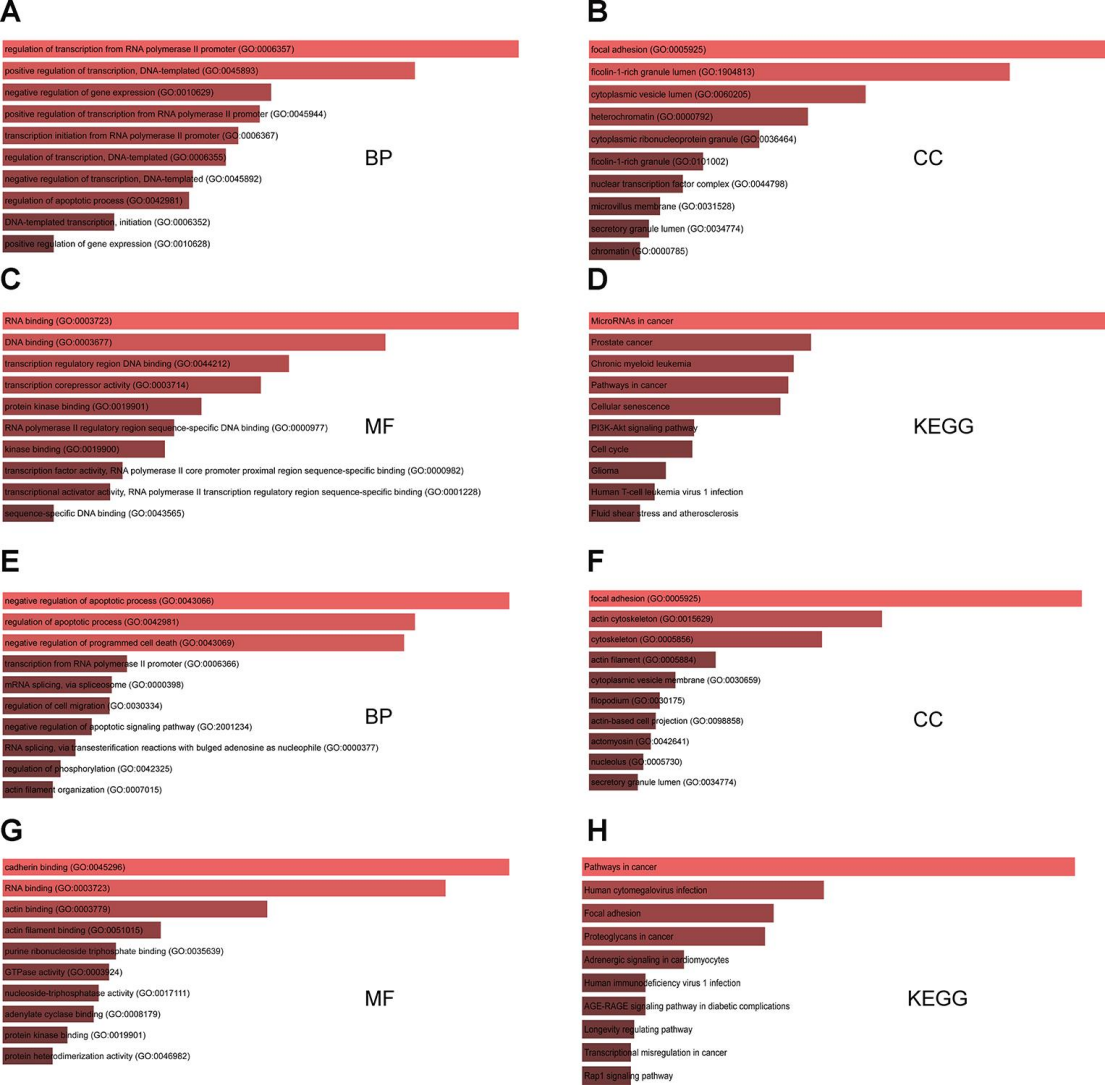
**GO and KEGG analysis of genes targeted by DEMs commonly shared between the two GEO datasets.** (**A**–**C**) Top 10 GO biological process (BP), cellular component (CC), and molecular function (MF) terms enriched in target genes of the three upregulated miRNAs. (**D**) Top 10 KEGG pathways enriched in target genes of the three upregulated miRNAs. (**E**–**G**) Top 10 GO BP, CC, and MF terms enriched in target genes of the five downregulated miRNAs. (**H**) Top 10 KEGG pathways enriched in target genes of the five downregulated miRNAs.

GO functional annotation analysis results indicated that for targets of the three upregulated miRNAs the most enriched terms were ‘regulation of transcription from RNA polymerase II promoter’ and ‘positive regulation of transcription’ in the biological process (BP) category, ‘focal adhesion’ and ‘ficolin-1-rich granule lumen’ in the cellular component (CC) category, and ‘RNA and DNA binding’ in the molecular function (MF) category (Figure 2A–2C). In turn, the target genes of the five downregulated miRNAs were mostly enriched in ‘negative regulation of apoptosis’ and ‘programmed cell death’ in the BP category, ‘focal adhesion’ and ‘actin cytoskeleton’ in the CC category, and ‘cadherin binding’ and ‘RNA binding’ in the MF category (Figure 2E–2G). KEGG pathway analysis showed that for targets of the upregulated miRNAs the most enriched pathways were ‘microRNAs in cancer’, ‘phosphoinositide 3-kinase (PI3K)-AKT signaling pathway’, and ‘cell cycle’ (Figure 2D). For targets of the downregulated miRNAs, the most enriched pathways were ‘cancer’, ‘focal adhesion’, and ‘proteoglycans in cancer’ (Figure 2H).

### Construction of target gene-PPI and miRNA-hub gene networks

**Figure 3:**
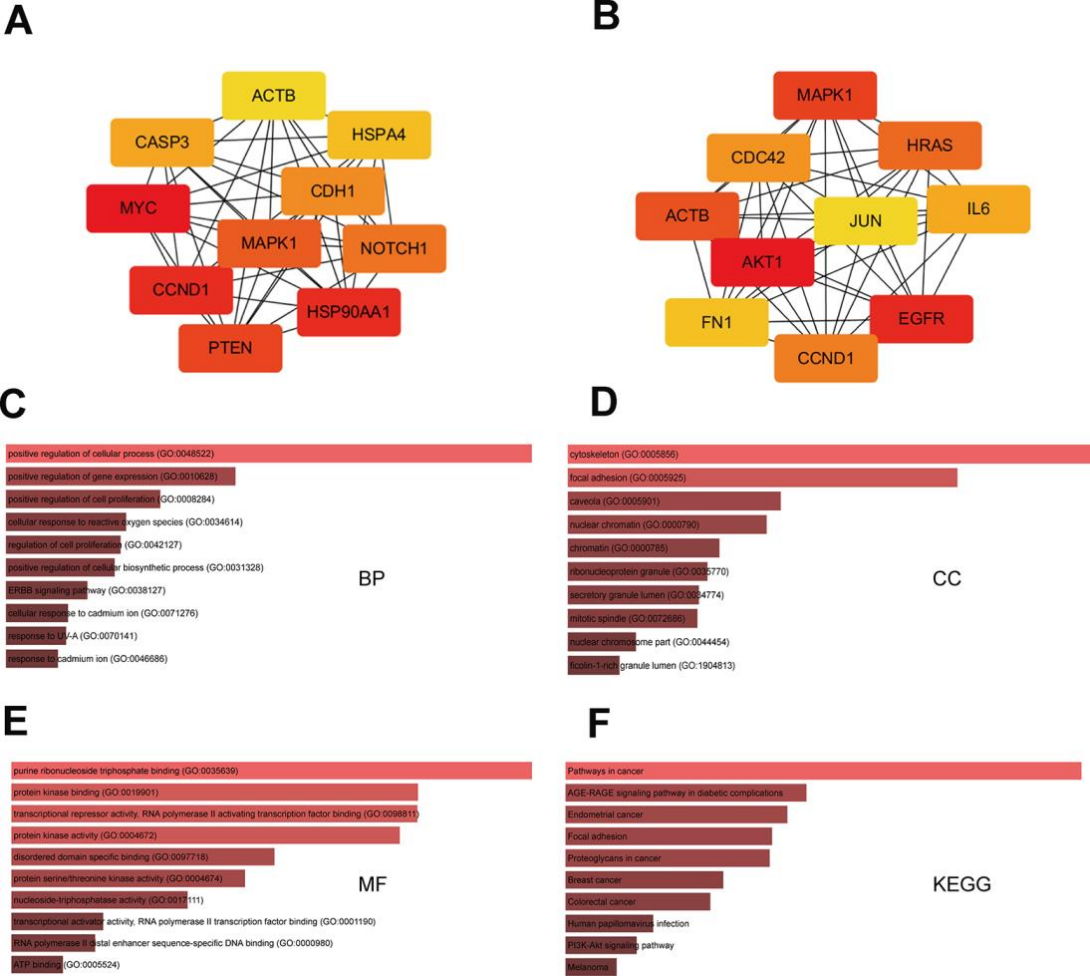
**GO and KEGG analysis of hub-gene targets.** (**A**) Top 10 hub-gene targets for the three upregulated miRNAs. (**B**) Top 10 hub-gene targets for the five downregulated miRNAs. (**C**–**E**) Top 10 GO BP, CC, and MF terms enriched in the top 20 hub-gene targets. (**F**) Top 10 KEGG pathways enriched in the 20 hub-gene targets.

**Figure 4:**
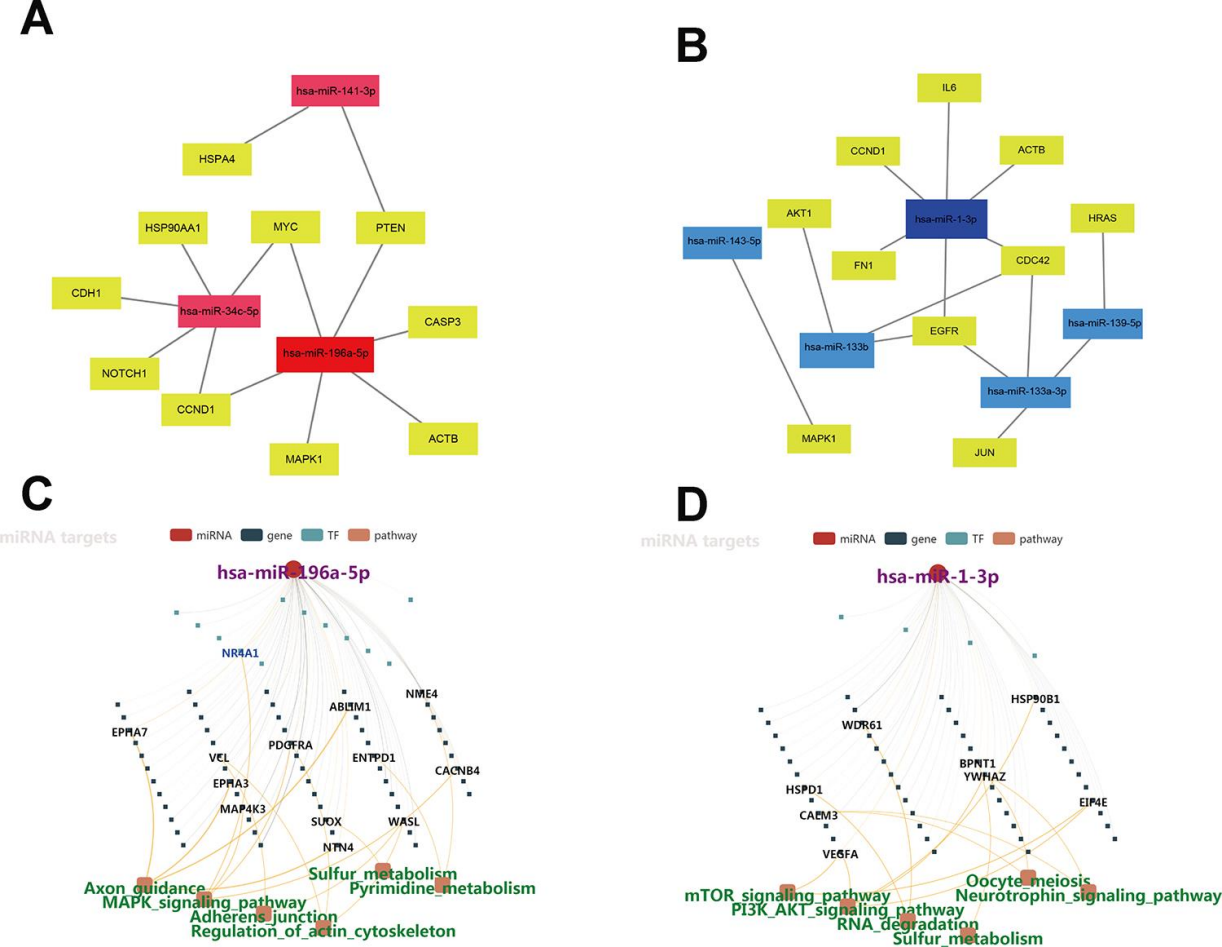
**Regulatory networks of miRNAs-hub-gene targets.** (**A**) Interaction network of the three upregulated miRNAs and their hub-gene targets. (**B**) Regulatory network of the five downregulated miRNAs and their hub-gene targets. (**C**–**D**) Regulatory networks of miR-196a-5p and miR-1-3p and associated signaling pathways.

To identify hub genes among the targets of the eight DEMs defined above, PPI networks were constructed using the STRING database and Cytoscape software [15]. The top 10 hub genes thus identified are shown in Figure 3A and 3B. For the three upregulated miRNAs, the predicted hub genes were *MYC, CCND1, HSP90AA1, PTEN, MAPK1, NOTCH1, CDH1, CASP3, HSPA4,* and *ACTB*. For the five downregulated miRNAs, the predicted hub genes were *AKT1, CCND1, CDC42, IL6, FN1, MAPK1, JUN, EGFR, ACTB,* and *HRAS*. To verify potential importance in the development of ESCC, GO and KEGG analyses were also performed on these gene clusters. Results showed that these genes were significantly enriched in various GO functions, such as ‘positive regulation of cellular process’ and ‘positive regulation of cell proliferation’ in the BP category (Figure 3C), ‘cytoskeleton’ and ‘focal adhesion’ in the CC category (Figure 3D), and ‘purine ribonucleoside triphosphate binding’ and ‘protein kinase binding’ in the MF category (Figure 3E). These genes were also enriched in prominent cancer-related signaling pathways (Figure 3F). Subsequently, a miRNA-hub gene network was constructed using Cytoscape software. Interactional analysis indicated that among the 10 hub genes targeted by the upregulated miRNAs, six (*MYC, ATCB, CCND1, MAPK1, PTEN,* and *CASP3*) are potentially regulated by miR-196a-5p (Figure 4A), while another six (*IL6, CCND1, ATCB, CDC42, EGFR,* and *FN1*) are potentially regulated by miR-1-3p (Figure 4B). Utilizing the miRNACancerMAP database, we surveyed cellular signaling pathways involving miR-196a-5p and miR-1-3p. Results showed association of these miRNAs with signaling cascades closely related to tumor growth, including MAPK, adherens junction, mechanistic target of rapamycin (mTOR), and PI3K-AKT pathways (Figure 4C and 4D). Since these results overlapped with those obtained in our pathway analysis, we deemed that miR-196a-5p and miR-1-3p may be essential miRNAs in ESCC pathogenesis.

**Figure 5:**
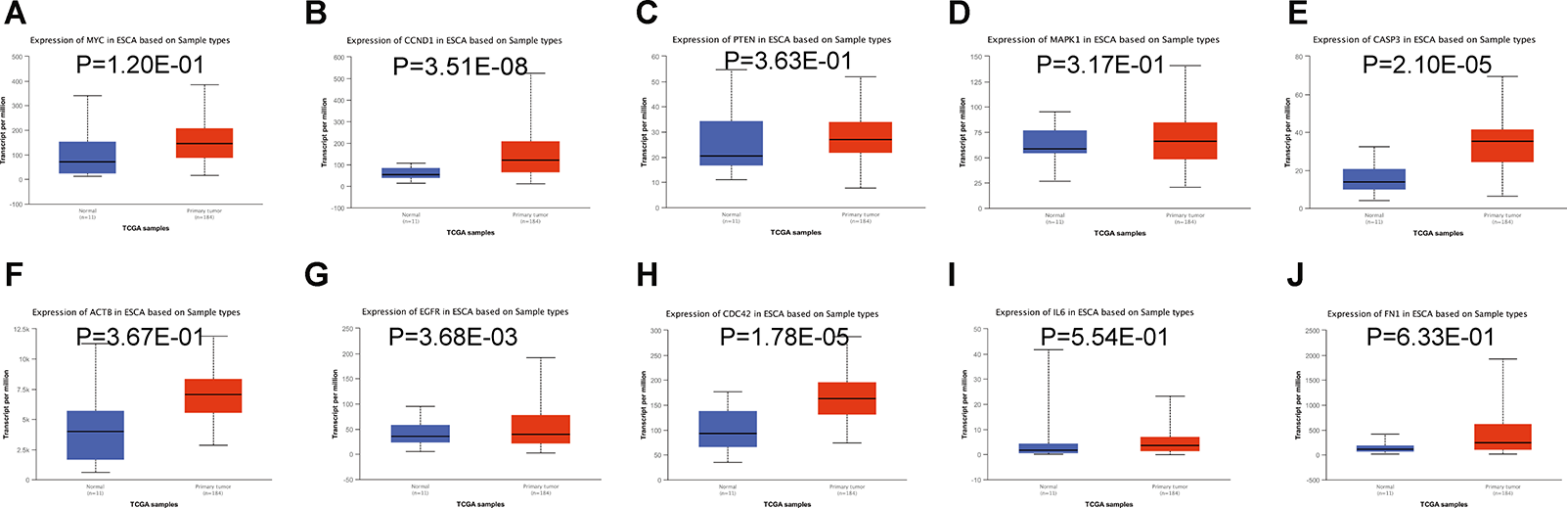
**Relative expression of hub-gene targets of miR-196a-5p and miR-1-3p.** (**A**–**E**) Relative expression of *MYC, CCND1, PTEN, MAPK1,* and *CASP3* mRNAs in esophageal carcinoma, compared to normal esophageal tissue samples. (**F**–**J**) Relative expression of *ACTB, EGFR, CDC42, IL6,* and *FN1* mRNAs in esophageal carcinoma, compared to normal esophageal tissue samples. Analysis of esophageal carcinoma RNA-seq datasets in TCGA was performed on the UALCAN database.

Subsequently, we accessed the UALCAN tool to assess within TCGA the expression of the most relevant target genes of miR-196a-5p and miR-1-3p in esophageal carcinoma [16]. Results indicated that the expression of four target genes (*CCND1, CASP3, EGFR,* and *CDC42*) was markedly upregulated in esophageal carcinoma (Figure 5B, 5E, 5G, and 5H). Since sampling size was relatively limited, further verification may still reveal differential expression of other hub genes regulated by miR-196a-5p and miR-1-3p in ESCC.

### Prognostic value of miR-196a-5p and miR-1-3p expression in ESCC

**Figure 6:**
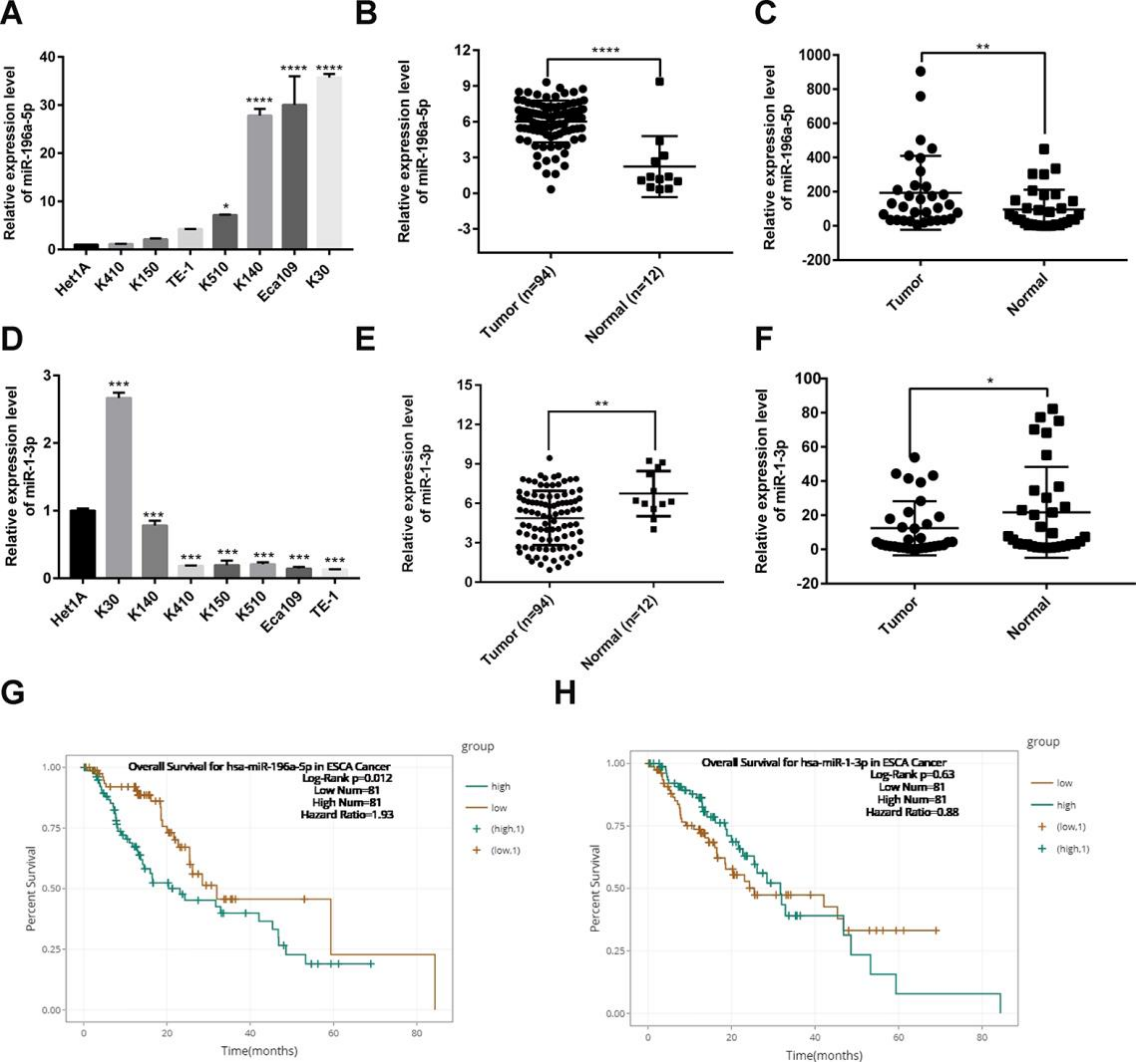
**Expression analysis and prognostic value of miR-196-5p and miR-1-3p in ESCC.** (**A**) Analysis of miR-196a-5p expression by qPCR in ESCC and normal esophageal epithelial (Het1A) cells. (**B**) Expression of miR-196a-5p in ESCC and normal esophagus samples from TCGA. (**C**) Expression of miR-196a-5p in ESCC and paired normal tissue samples (n = 32) collected at our institution. (**D**) Expression of miR-1-3p in ESCC and Het1A cells. (**E**) Expression of miR-1-3p in ESCC and normal esophagus samples from TCGA. (**F**) Expression of miR-1-3p in ESCC samples and paired normal tissue samples (n = 32) collected at our institution. (**G** and **H**) Kaplan-Meier survival analysis based on miR-196a-5p and miR-1-3p expression in esophageal carcinoma samples from TCGA. *P < 0.05, **P < 0.01. ***P < 0.001, and ****P < 0.0001.

To complement the above bioinformatics analyses, we verified the expression of miR-196a-5p and miR-1-3p in ESCC cell lines, the TCGA database, and clinical ESCC cases. Using qPCR, we determined that either upregulation or downregulation of these two hub DEMs occurred in different ESCC cell lines (KYSE30, KYSE140, KYSE410, KYSE150, KYSE510, Eca109, and TE-1), compared to normal esophageal epithelial (Het1A) cells (Figure 6A and 6D). Analysis of miRNA expression profiles from TCGA showed that the expression of miR-196a-5p was significantly higher, whereas that of miR-1-3p was significantly lower, in ESCC samples compared to normal esophageal tissue (Figure 6B and 6E). In addition, we collected 32 ESCC samples from our institution and summarized their clinicopathological data. Results from qPCR assays confirmed that both miR-196a-5p and miR-1-3p were differentially expressed in ESCC samples (Figure 6C and 6F). Analysis of the relationship between miR-196a-5p and miR-1-3p expression and clinicopathological features showed that the expression of these two miRNAs was associated with T classification in ESCC patients (Supplementary Tables 1–3). Meanwhile, survival analysis of TCGA data on the starBase database [17], showed that low miR-196a-5p expression was associated with better prognosis in patients with esophageal carcinoma. In contrast, no prognostic significance was found for miR-1-3p (Figure 6G and 6H).

### miR-196a-5p and miR-1-3p exert opposing roles in ESCC cell proliferation and migration

**Figure 7:**
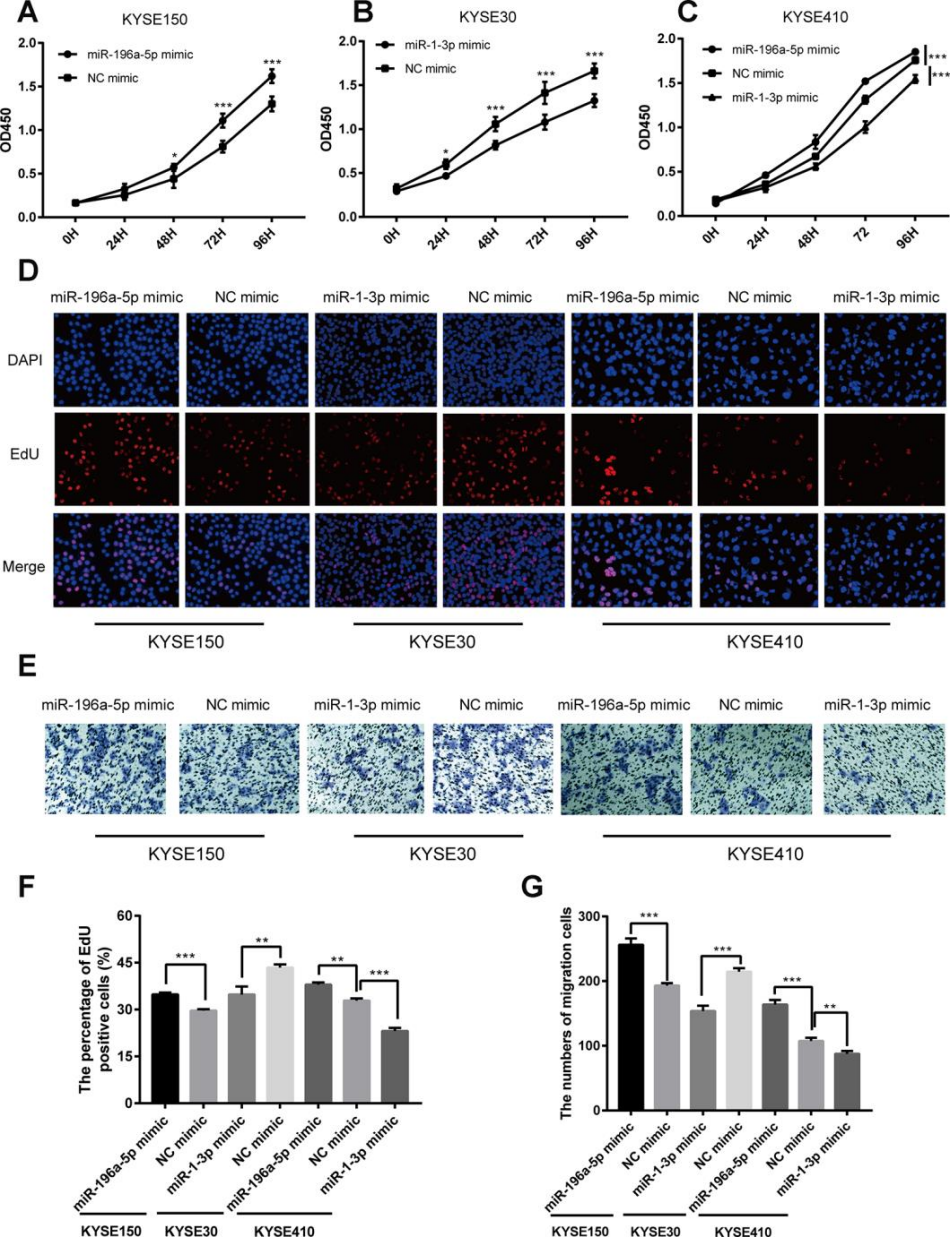
**Opposing effects of miR-196a-5p and miR-1-3p on proliferation and migration in cultured ESCC cells.** (**A**–**C**) CCK-8 cell proliferation assay results from KYSE150, KYSE30, and KYSE410 cells transfected with mimics of miR-196a-5p, miR-1-3p, or negative control (NC). (**D, F**) EdU cell proliferation assay results from KYSE150, KYSE30, and KYSE410 cells transfected with mimics of miR-196a-5p, miR-1-3p, or NC. (**E, G**) Transwell migration assay results from KYSE150, KYSE30, and KYSE410 cells transfected with miRNA mimics. *P < 0.05, **P < 0.01, and ***P < 0.001.

Next, we sought to elucidate the functional effects of miR-196a-5p and miR-1-3p dysregulation through studies on ESCC cells in vitro. To this end, we transfected ESCC cell lines with miR-196a-5p and miR-1-3p mimics or their negative controls (NC). Considering transfection efficiency and background expression of the two miRNAs, we upregulated miR-196a-5p in KYSE150 and KYSE410 cells and miR-1-3p in KYSE30 and KYSE410 cells. Subsequently, cell proliferation was measured using Cell Counting Kit-8 (CCK-8) and 5-ethynyl-2-deoxyuridine (EdU) assays. As shown in Figure 7A–7C (CCK-8 results), Figure 7D and 7F (EdU results), compared to the corresponding NCs, upregulation of miR-196a-5p promoted ESCC cell proliferation whereas upregulation of miR-1-3p showed inhibitory effects. We next assessed the impact of miR-196a-5p and miR-1-3p upregulation on the migrational potential of ESCC cells using Transwell migration assays. Results showed that upregulation of miR-196a-5p enhanced, whereas upregulation of miR-1-3p markedly suppressed, the migrational ability of the ESCC cell lines tested (Figure 7E and 7G).

## DISCUSSION

ESCC is the most common form of esophageal cancer in developing countries, and its incidence is particularly high in the so-called "Asian esophageal cancer belt", an area traversing northern China, southern Russia, north-eastern Iran, northern Afghanistan, and eastern Turkey [18, 19]. At advanced stages, ESCC forms large, life-threatening tumors that block the esophagus and prevent patients from eating and drinking normally. Although marked advances have been achieved in treating ESCC, the 5-year overall survival rate of ESCC is still less than 20%, and lower than 5% for patients with distant metastases [20]. Thus, identifying novel, more effective diagnostic and treatment targets is urgently needed.

MicroRNAs are small ncRNA molecules that regulate target genes and downstream signaling pathways by post-transcriptional mechanisms. MiRNAs are critical functional molecules in tumor progression [3, 21, 22] and have in consequence attracted great interest as potential therapeutic targets [23–25]. However, because there are typically numerous miRNAs influencing cancer cells’ activities, choosing the most suitable target(s) for tumor diagnosis and treatment remains a significant challenge.

The role of bioinformatics in the exploration of disease mechanisms has become essential given the multifactorial nature of many common health conditions. Examples include the identification of miRNAs associated with breast cancer recurrence and hub genes involved in plaque deterioration in atherosclerosis [26, 27]. In the present study, we analyzed miRNA expression profiles from two GEO microarray datasets containing ESCC and normal esophageal tissue samples, and obtained eight miRNAs (three up-regulated and five down-regulated), shared by both miRNA datasets, with differential expression in ESCC. Following detection of potential target mRNAs for these DEMs in the miRTarBase database, GO annotation and KEGG pathway enrichment analyses of the target gene lists revealed their participation in several cellular processes (e.g. RNA and DNA binding, focal adhesion, and regulation of apoptosis) and signaling cascades related to tumor development (e.g. cell cycle, MAPK, and PI3K-AKT pathways). A PPI network based on these genes was next constructed to obtain the top 10 hub genes, and subsequent analysis confirmed their involvement in important tumor-related pathways. Examples included *CCND1* and *EGFR*, i.e. key regulators of cell cycle progression and PI3K-AKT signaling in a variety of normal and cancer tissues [28, 29]. Subsequently, a miRNA-target gene interaction network revealed an extensive regulatory role for miR-196a-5p and miR-1-3p, which are respectively upregulated and downregulated in ESCC, as each of these miRNAs was predicted to control the activity of 6 of the top 10 hub genes in the DEM-gene network. Expression analysis of miR-196a-5p and miR-1-3p targets in TCGA using the UALCAN tool showed that *CCND1, CASP3, EGFR,* and *CDC42* were overexpressed in esophageal carcinoma, compared to normal esophagus samples. *CCND1* has been revealed as a miR-1 target in neonatal cardiomyocytes [30] while we speculate that *CASP3* expression might be regulated by other RNA species such as lncRNAs or circRNAs.

The results of these bioinformatics analysis were contrasted with qPCR data and correlation analyses in 32 ESCC cases from our institution. In line with in silico data mining results, we found that miR-196a-5p was overexpressed and miR-1-3p was underexpressed in tumor samples, compared with matched control specimens. Although these differences were significant, actual expression changes were not too large, thus verification in a larger sample is needed in the future. Analysis of the association between miR-196a-5p and miR-1-3p expression and clinicopathologic features showed a correlation between these miRNAs and ESCC T classification, which suggests that changes in miR-196a-5p and miR-1-3p expression may impact tumor progression. Data mining of TCGA database also confirmed expression differences for both miRNAs in ESCC, and showed that high miR-196a-5p expression correlated with poorer prognosis. Although TCGA analysis results did not show an association between miR-1-3p expression and prognosis in patients with ESCC, several studies indicated that high miR-1-3p expression is associated with better prognosis in ESCC [31–33]. Indeed, previous studies have highlighted a number of biological actions and clinicopathological associations of miR-196a-5p and miR-1-3p in patients with ESCC [34–36]. Recent work also showed that several miRNAs, including miR-200b-3p, miR-31-5p, miR-15b-5p, miR-141-3p, miR-135b-5p, and miR-195-5p, were correlated with overall survival of ESCC patients.

Although similar (i.e. opposing) expression trends for miR-196a-5p and miR-1-3p were found in most ESCC cell lines tested by us, miR-1-3p showed instead a relatively high expression in KYSE30 cells. The reason for this exception is unclear but may reflect a distinct genetic background in this cell line. We tested the effects of overexpressing miR-196a-5p and miR-1-3p on cell proliferation and migration and found that miR-196a-5p enhances, while miR-1-3p reduces, proliferative and migration potential in ESCC cell lines. To our surprise, these effects were also observed in KYSE30 cells, which showed as mentioned relatively high baseline miR-1-3p expression.

There are some limitations in our study. We did not assess proliferation and migration after knocking down miR-196a-5p and miR-1-3p, and insufficient clinical sampling size precluded more accurate assessment of the effects of these miRNAs on clinicopathological variables and outcomes of our ESCC cohort. We did not prove that miR-196a-5p and miR-1-3p are more important than other miRNAs through further experiments. In addition, the influence of other non-coding RNAs, mainly lncRNAs and circRNAs, on ESCC pathogenesis were not addressed and deserve further scrutiny. Although the novelty of our findings is somewhat reduced in light of several publications demonstrating a pro-oncogenic role for miR-196a-5p and a tumor-suppressor role for miR-1-3p in various cancer types, our data shed light on the complex landscape of ESCC regulation by ncRNAs by highlighting through miRNA-hub gene network analysis previously undefined interactions that potentially affect ESCC development and metastasis.

In conclusion, our findings strongly suggest that miR-196a-5p upregulation and miR-1-3p downregulation impact tumor stage and patient survival by concurrently promoting proliferation and migration in ESCC cells. Although further research is warranted, the evidence gathered so far suggests that these miRNAs might be novel co-therapeutic targets for the diagnosis and treatment of ESCC.

## MATERIALS AND METHODS

### Data collection and study design

Two ESCC miRNA expression microarray datasets (GSE114110 and GSE43732) were retrieved from the GEO repository (https://www.ncbi.nlm.nih.gov/geo). GSE114110 included 30 ESCC and 10 normal esophageal epithelial samples [12], while GSE43732 included 119 ESCC samples and paired adjacent normal tissues [13]. All samples were collected in China and originated from patients who had not received anticancer treatment. The above datasets were produced independently using the GPL24967 and GPL16543 platforms, respectively. Therefore, we normalized these data using R’s limma package.

### DEM screening and Venn diagram analysis

After obtaining standardized chip data from the GSE114110 and GSE43732 datasets, differential miRNA expression in ESCC was investigated in relation to normal esophageal tissue (control) using the limma software package in R. We set *P* < 0.05 and |log2 fold change (FC)| ≥ 1 as the thresholds to identify DEMs. Overlapping miRNAs in the two datasets were screened using Venn diagram analysis with the VennDiagram R package [37].

### Prediction of target genes and miRNA pathway analysis

MiRTarBase, an experimentally validated database of miRNA-target interactions, was used to predict potential target genes for the eight overlapping DEMs. MiRNACancerMAP database was used to perform miRNA pathway analysis.

### GO and KEGG pathway analyses

Functional and pathway analyses of the predicted target genes of the eight overlapping DEMs were conducted using the GO and KEGG databases, respectively, by processing data with the DAVID online tool (https://david.ncifcrf.gov/) [38]. *P* < 0.05 was considered significant.

### Construction of target gene-PPI and miRNA-gene networks

To assess the functional associations among the target genes of upregulated and downregulated DEMs, we uploaded target gene data to the STRING database. Interactions with a combined score > 0.4 were considered significant. Highly interconnected (hub) genes in the PPI network were analyzed using Cytoscape software (version 3.6.0). After hub DEMs and hub target genes were identified, Cytoscape was used to visualize the resulting miRNA-gene network.

### Differential gene expression and survival analyses

After extracting expression data for miR-196a-5p and miR-1-3p from ESCC and normal esophageal epithelial tissues from the TCGA database, analysis of differential expression of target genes was performed on the University of Alabama Cancer Database (UALCAN; http://ualcan.path.uab.edu/) resource. The StarBase database was used to evaluate the prognostic values of hub miRNAs in esophageal carcinoma through Kaplan-Meier plots.

### Cell lines and clinical samples

Human ESCC KYSE30, KYSE140, KYSE410, KYSE150, KYSE510, Eca109, and TE-1 and normal esophageal epithelial (Het1A) cell lines were cultured in RPMI-1640 medium supplemented with 10% fetal bovine serum (FBS; BI, Israel), 100 U/mL penicillin, and 100 μg/mL streptomycin at 37°C in an incubator with a humidified atmosphere containing 5% CO_2_.

Thirty-two ESCC and paired normal adjacent tissue samples were acquired from patients undergoing surgical procedures at the Third Affiliated Hospital of Soochow University. Patient baseline characteristics were collected from medical records. All samples were obtained after written informed consent was provided, in accordance with the Code of Ethics of the World Medical Association (Declaration of Helsinki). All protocols for the use of patient samples in this study were approved by the Ethics Committee of the Third Affiliated Hospital of Soochow University.

### Cell transfection

Negative control (NC), miR-196a-5p, and miR-1-3p mimics were purchased from GenePharma (Suzhou, China), and transfected into ESCC cell lines using siRNA-Mate (GenePharma) according to the manufacturer’s instructions.

### RNA extraction and quantitative PCR

TRIzol reagent was used to extract RNA from clinical samples and ESCC cell lines. The miRNAs first-strand cDNA and q-PCR kits were purchased from TIANGEN (Beijing, China) and U6 snRNA was used as the internal reference. The forward primers used were: miR-196a-5p: 5'-CGCGTAGGTAGTTTCATGTTGTTGGG-3, miR-1-3p: 5'-GCGCGCTGGAATGTAAAGAAGTATGTAT-3', and U6 snRNA: 5'-CTCGCTTCGGCAGCACA-3'. The reverse primers were provided in the qPCR detection kit. Relative miRNA expression levels were calculated using the 2^-ΔΔCt^ method.

### Cell proliferation assay

The CCK-8 assay (Dojindo, Kumamoto, Japan) was used to quantify cell proliferation. Twenty-four h after miRNA mimic transfection, cells were seeded into 96-well culture plates (1.5 × 10^3^ cells/well). At different time points, 10 μL CCK-8 solution was added to each well, incubated for 2 h, and samples’ optical density (OD) was measured immediately at 450 nm. The Cell-Light EdU Apollo567 In Vitro Kit (Cat.C10310-1, Ruibo Biotech., Guangzhou, China) was also used to assess cell proliferation. To this end, cells transfected with miRNA mimics for 24 h were seeded into 96-well plates (1.5 × 10^4^ cells/well). After 24 h, the cells were incubated in EdU working solution for 2 h, fixed with 4% paraformaldehyde, permeabilized, washed, and stained with 1x Apollo solution according to the manufacturer’s instructions. Results were analyzed from microphotographs taken using a fluorescence microscope.

### Transwell migration assay

Transwell inserts were used to conduct migration assays. After transfection, cells were resuspended in serum-free culture solution and approximately 3 × 10^4^ cells were loaded into the upper Transwell chamber. The lower chamber was filled with 600 μL of culture medium containing 20% FBS. After 24 h, the cells that remained on the upper chamber were removed and those that migrated through the membrane were fixed in 4% paraformaldehyde for 30 min, stained with 0.1% crystal violet, and counted in five randomly selected fields under an inverted microscope.

### Statistical analysis

GraphPad Prism 7 software was used to perform unpaired Student’s *t*-tests to analyze differences between two groups. *P* < 0.05 was regarded as significant.

## Supplementary Material

Supplementary Tables
