# Supplementary material for: Identification and validation of hub microRNAs dysregulated in esophageal squamous cell carcinoma
Source: Aging (Albany NY). 2020 May 15;12(10):9807–24. doi: 10.18632/aging.103245 (PMC7288914; doi:10.18632/aging.103245)
Supplement: Supplementary Tables [file aging-12-103245-s001..pdf]

SUPPLEMENTARY TABLES

Supplementary Table 1. Clinicopathological characteristics of ESCC patient samples (n=32).

Characteritiscs	No. of cases (%)
**Age (y)**	
<65	15 (46.9)
≥65	17 (53.1)
**Gender**	
Male	28 (87.5)
Female	4 (12.5)
**TNM stage**	
I	6 (18.8)
II	13 (40.6)
III	13 (40.6)
**T classification**	
T1	5 (15.6)
T2	9 (28.1)
T3	18 (56.3)
T4	0 (0)
**N classification**	
N0	12 (37.5)
N1	17 (53.1)
N2-NX	3 (9.4)
**Metastasis**	
No	32 (100)
Yes	0 (0)
**Vascular invasion**	
No	25 (78.1)
Yes	7 (21.9)
**Lymphatic invasion**	
No	13 (40.6)
Yes	19 (59.4)
**Expression of miR-221**	
Low expression	16 (50)
High expression	16 (50)

Supplementary Table 2. Correlation between miR-196a-5p expression and clinicopathologic characteristics of ESCC patients (n=32).

Characteritiscs	miR-196a-5p		p value
	Low expression, no. cases	High expression, no. cases	
**Age(y)**			
<65	7	8	0.723
≥65	9	8	
**Gender**			
Male	15	13	0.285
Female	1	3	
**TNM stage**			
I	2	4	0.488
II	8	5	
III	6	7	
**T classification**			
T1	5	0	**0.020**
T2	2	7	
T3	9	9	
T4	0	0	
**N classification**			
N0	7	5	0.696
N1	8	9	
N2-NX	1	2	
**Metastasis**			
No	20	12	—
Yes	0	0	
**Vascular invasion**			
No	14	11	0.200
Yes	2	5	
**Lymphatic invasion**			
No	7	6	0.719
Yes	9	10	

Supplementary Table 3. Correlation between miR-1-3p expression and clinicopathologic characteristics of ESCC patients (n=32).

**Characteritiscs**	**miR-1-3p**		**p value**
	**Low expression, no. cases**	**High expression, no. cases**	
**Age(y)**			
<65	9	6	0.288
≥65	7	10	
**Gender**			
Male	15	13	0.285
Female	1	3	
**TNM stage**			
I	2	4	
II	7	6	0.663
III	7	6	
**T classification**			
T1	9	9	
T2	2	7	**0.020**
T3	5	0	
T4	0	0	
**N classification**			
N0	6	6	
N1	8	9	0.822
N2-NX	2	1	
**Metastasis**			
No	16	16	—
Yes	0	0	
**Vascular invasion**			
No	14	11	0.200
Yes	2	5	
**Lymphatic invasion**			
No	6	7	0.719
Yes	10	9	
